# Association Between Adherence to the Dietary Approaches to Stop Hypertension (DASH) Diet and Diminished Ovarian Reserve: A Case–Control Study

**DOI:** 10.1002/hsr2.73457

**Published:** 2026-11-03

**Authors:** Mahdieh Khodarahmi, Amin Mokari‐Yamchi, Mahdi Vajdi, Sanaz Mehrabani, Hatav Ghasemi‐Tehrani, Gholamreza Askari, Abed Ghavami

**Affiliations:** ^1^ Nutrition and Food Security Research Center Isfahan University of Medical Sciences Isfahan Iran; ^2^ Maternal and Childhood Obesity Research Center Urmia University of Medical Sciences Urmia Iran; ^3^ Nutrition and Food Security Research Center, Department of Community Nutrition, School of Nutrition and Food Science Isfahan University of Medical Sciences Isfahan Iran; ^4^ Department of Obstetrics and Gynecology, School of Medicine Isfahan University of Medical Sciences Isfahan Iran

**Keywords:** anti‐mullerian hormone, antral follicle count, dietary quality, diminished ovarian reserve, infertility

## Abstract

**Background:**

Despite the fact that dietary intakes are suggested to affect fecundity and reproductive outcomes, data about the favorable impacts of dietary quality indicators on diminished ovarian reserve (DOR) is scarce. So, we aimed to examine the relationship between the Dietary Approaches to Stop Hypertension (DASH) score and odds of DOR.

**Methods:**

This case‐control study was conducted among 370 women (120 with DOR as the case group and 250 with normal ovarian reserve as the control group). Cases and controls were frequency‐matched according to age and body mass index (BMI) at the study‐design stage. Dietary information derived from a validated semi‐quantitative food frequency questionnaire (FFQ) was used to count energy‐adjusted DASH score based on eight components. Markers of ovarian reserve, including anti‐Müllerian hormone (AMH) and antral follicle count (AFC) which were measured in serum samples and by transvaginal ultrasound, respectively. The multivariable‐adjusted binary logistic regression was used to identify the association between energy‐adjusted DASH score and odds of DOR.

**Results:**

Higher energy‐adjusted DASH score was related to lower diastolic blood pressure (DBP) in women with DOR (*p* = 0.006). No statistically significant association was observed between DASH adherence and the odds of DOR. In the crude model, compared with the lowest tertile (T1), the odds ratios for DOR were 0.87 (95% CI: 0.51–1.49) for T2 and 0.78 (95% CI: 0.46–1.33) for T3 (P‐trend = 0.370). After adjustment for potential confounders, the association remained non‐significant in Model 1 (T2: 0.93, 95% CI: 0.53–1.63; T3: 0.76, 95% CI: 0.43–1.33; P‐trend = 0.328) and Model 2 (T2: 0.88, 95% CI: 0.49–1.56; T3: 0.74, 95% CI: 0.42–1.30; P‐trend = 0.288). Nevertheless, in an exploratory unadjusted analysis among women with DOR, AFC differed significantly across energy‐adjusted DASH tertiles, with higher AFC observed among women in the higher DASH adherence categories (*p* = 0.015).

**Conclusions:**

Higher adherence to the DASH dietary pattern was not significantly associated with the odds of DOR. Further prospective studies are warranted to clarify the potential relationship between dietary patterns and ovarian reserve.

## 1 Introduction

Diminished ovarian reserve (DOR) is a condition characterized by a reduction in the quantity or quality of oocytes in the ovary in women of reproductive age [1], which is accompanied by a decrease in serum anti‐Müllerian hormone (AMH) and antral follicle count levels [2]. It is estimated that DOR affects approximately 10%–26% of women during their reproductive lifetime [3]. It has been reported that women with DOR have a higher propensity for decreased fecundity, inadequate response to infertility treatments, and early menopause, which severely influence both reproductive and nonreproductive health status [4, 5]. DOR, as a complex clinical phenomenon, can be influenced by some well‐known factors like gynecological conditions (such as endometriosis, tumors, pelvic infections, etc.), genetic alterations, and advanced age [6, 7]. Although usage of assisted reproductive technologies [8] has been shown to treat infertility, the success rate of that is limited (30%–40%) [9]. This modest success rate along with high costs [10], adverse outcomes for babies [11], and restricted regional availability of infertility treatments [12], encourage the requirements to determine the role of modifiable risk factors in DOR and the prevention of this condition so as to prolong the woman's reproductive lifetime.

It has been hypothesized that diet has the capability to modify the rate of fall in ovarian reserve [13]. There are some epidemiological studies that have focused on the relations of nutritional components, including nutrients and food groups, with female fertility and ovulation [14]. The potentially favorable dietary ingredients include low glycemic index (GI) carbohydrates, fiber, omega‐3 fatty acids, vitamin D, and antioxidants, which have been shown to delay ovarian aging. Conversely, excessive amounts of animal‐derived protein, trans fatty acids (TFAs), saturated fatty acids (SFAs), and high GI foods negatively influence fertility [14]. Undoubtedly, most of this evidence is based on individual nutrients or specific foods as opposed to dietary patterns assessing the effects of diet comprehensively, which provide better prediction of overall health condition [15]. Accordingly, dietary indicators that have been developed based on current nutritional knowledge and measure compliance with dietary guidelines tend to be more practical for developing standardized dietary recommendations [16].

The Dietary Approaches to Stop Hypertension (DASH) score, a scale to measure adherence to the DASH diet, is characterized by high intake of fruits, vegetables, nuts, legumes, wholegrains and low‐fat dairy products and with a low consumption of animal protein, sugar, SFAs and sodium, have been shown to exert favorable effects on markers of insulin metabolism, biomarkers of inflammation and oxidative stress, lipid homeostasis and weight managements that is essential in the context of fertility [17]. Besides, prior studies have indicated that the DASH diet, owing to its high content of micronutrients (magnesium, calcium, folate, and fiber), antioxidants, phytoestrogens, isoflavones, accompanied by its low GI, might improve metabolic profile in polycystic ovary syndrome (PCOS) [18], which has been accounted for about 75% of anovulatory infertility cases [8]. However, on the basis of the current knowledge, there is considerable uncertainty regarding the relationship of the a priori dietary indices, particularly the DASH score, with ovarian reserve markers, including AFC and AMH levels. Accordingly, whether dietary factors are associated with the odds of DOR remains unclear. Therefore, we conducted a case‐control study to investigate the association between adherence to the DASH diet and DOR among women attending an infertility clinic.

## 2 Methods

### 2.1 Subjects

This case‐control study was conducted between July 2023 and December 2023 at the Infertility Clinic of Shahid Beheshti Women's Hospital, affiliated with Isfahan University of Medical Sciences, Isfahan, Iran. Women attending the infertility clinic for the evaluation of infertility or reproductive health concerns were consecutively screened for eligibility. The source population consisted of reproductive‐aged women referred to this clinic during the study period. Eligible participants were women aged 18–45 years, of Persian ethnicity, with a BMI of 20–35 kg/m^2^. The diagnosis of DOR was made by an experienced gynecologist (H.GHT) based on criteria that included low AMH levels (≤ 0.7 ng/mL), a low AFC (≤ 4 across both ovaries), or both [19]. Subjects with incomplete food frequency questionnaires (FFQ), as well as those with a history of ovarian surgery, chemotherapy, or radiotherapy, were excluded from the study. Additionally, individuals diagnosed with conditions such as endometriosis, premature ovarian failure, or various endocrine and metabolic disorders were not included. Participants who had received hormone therapy, adhered to specific dietary plans, or used oral contraceptives within 3 months prior to the study's start were also excluded. Controls with normal ovarian reserve were randomly selected during the same recruitment period from the same hospital, ensuring that both groups originated from the same source population. They did not meet the criteria for DOR, and therefore had AMH > 0.7 ng/mL and AFC > 4. Finally, 370 participants (250 controls, 120 with DOR were included in the analysis. The controls were frequency‐matched with cases by age (under 25, 25–30, and over 30 years) and BMI (under 24.9, 25–30, and over 30 kg/m^2^). Informed consent was provided by all of the participants, and the study protocol was approved by the Ethics Committee of Isfahan University of Medical Sciences (IR.ARI.MUI.REC.1401.297). This study followed the STROBE guideline [20]. Figure 1 shows the flowchart illustrating the study process.

**Figure 1:**
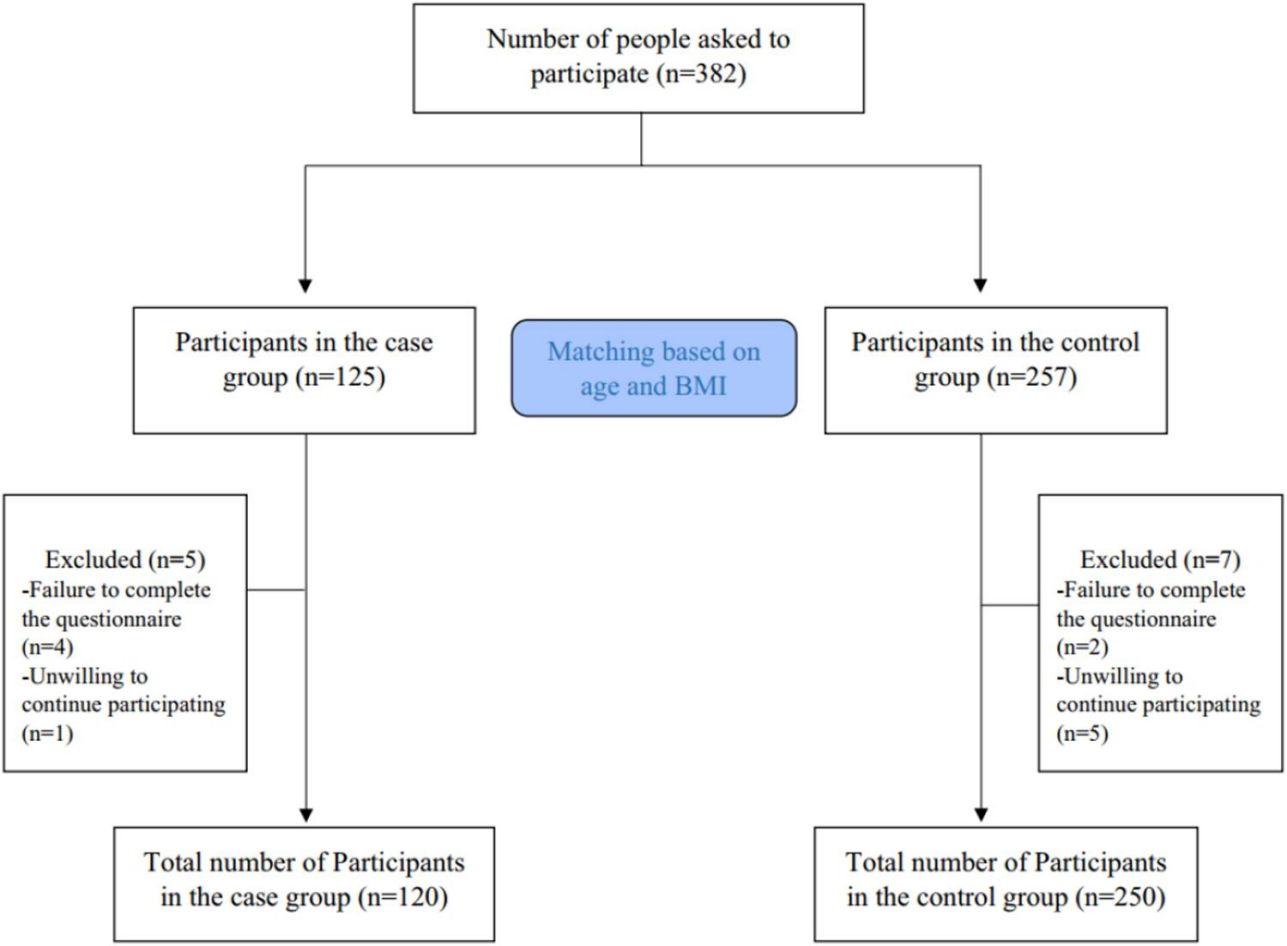
Flow chart of the study.

### 2.2 Dietary Assessment and Energy‐Adjusted DASH Score Calculation

Dietary intake was assessed during the study visit using a validated and reliable semi‐quantitative FFQ consisting of 80 food items [21]. The FFQ was designed to assess participants' habitual dietary intake over the preceding year. Participants were asked to indicate how often they consumed each item (daily, weekly, or monthly) based on standard serving sizes. Afterward, household measurements were utilized to transform the portion sizes of consumed food items into g/day using standard Iranian household measures [22]. Nutritionist IV software was then utilized to calculate daily energy and nutrient intake. While this software relies on the United States Department of Agriculture (USDA) food composition database, it has been adapted to include certain Iranian foods [23].

The dietary DASH score was assessed using the method developed by Fung [24]. Eight components included: higher intakes of whole grains, fruits, vegetables, legumes and nuts, and low‐fat dairy, and lower intakes of red or processed meats, sodium, and sweetened beverages. To account for the impact of individual energy intake on the DASH score components, each food component intake was adjusted to represent consumption per 1000 kcal. For each component, we classified participants into quintiles on the basis of their intake ranking. The component score for vegetables, fruits, nuts and legumes, dairy products, and whole grains and grain products was derived from the participant's quintile ranking (i.e., quintile 1 was assigned 1 point, while quintile 5 was assigned 5 points), in contrast to components of Na, red and processed meats, and sweetened beverages (5 points was assigned to quintile 1 whereas 1 point was assigned to quintile 5). We summed up the eight component scores to create an overall energy‐adjusted DASH score that ranged from 8 (minimum adherence) to 40 (maximal adherence).

The quintile cut‐offs for each DASH component were determined based on the distribution of the respective energy‐adjusted component intakes in the entire study population (*n* = 370), including both cases and controls. The overall energy‐adjusted DASH score was subsequently categorized into tertiles according to its distribution in the entire study population, with the lowest tertile representing the lowest adherence and the highest tertile representing the highest adherence to the DASH dietary pattern.

### 2.3 Anthropometric and Laboratory Parameters Assessment

Weight and height were measured while participants were wearing light clothing and without shoes to the nearest 100 grams and 0.5 centimeters (cm), respectively. BMI was calculated by dividing weight in kilograms by the square of height in meters. Waist circumference (WC) was measured with a non‐elastic tape, recorded to the nearest 0.1 cm. WC was measured at the midpoint between the lowest rib and the iliac crest, while hip circumference (HC) was taken at the widest point around the buttocks. The waist‐to‐hip ratio (WHR) was determined by dividing WC by HC. The average of the two readings was reported as the individual's SBP or DBP. International Physical Activity Questionnaire (IPAQ), which has been validated for the Iranian population was used to evaluate participants' daily physical activity by calculating the metabolic equivalent intensity [25]. Serum AMH levels were assessed using the enzyme‐linked immunosorbent assay [8] method (Monobind, California, USA), while the AFC in both ovaries was evaluated through transvaginal ultrasound on the third day of an unstimulated menstrual cycle.

### 2.4 Statistical Analyses

All statistical analyses were conducted using SPSS version 21.0 (SPSS Inc., Chicago, IL, 113 USA). Data were analyzed for normality using the Kolmogorov–Smirnov test and histogram charts. Baseline characteristics of the participants were expressed as mean ± standard deviation (SD) for quantitative variables and as frequencies (percentages) for qualitative variables. Independent sample *t*‐tests were applied to analyze the differences between cases and controls for continuous variables, whereas *χ* ^2^ tests were used for categorical variables. The overall energy‐adjusted DASH score was categorized into tertiles based on its distribution in the entire study population. The differences between participants across the tertiles of DASH score were compared using analysis of variance (ANOVA) and the *χ* ^2^ tests for quantitative variables and qualitative variables, respectively. Logistic regression was applied to estimate odds ratios (ORs) and 95% confidence intervals (CIs) for odds of DOR across energy‐adjusted DASH score tertiles. The analysis was adjusted for potential confounders, including Model I adjusted for age, BMI and physical, while Model II included Model I + FM and energy intake. Given the residual difference in BMI observed between cases and controls after frequency matching, BMI was retained in the multivariable models to account for potential residual confounding. Multicollinearity between BMI and fat mass was assessed using the variance inflation factor (VIF). The VIF value was 4.58, indicating no substantial multicollinearity between these variables. To assess the robustness of the findings and address potential socioeconomic confounding, sensitivity analyses were performed by additionally adjusting both multivariable models for education and occupation.

## 3 Results

Table 1 demonstrates the main characteristics of cases and controls. There was no statistically significant difference among participants (DOR and controls) in relation to age, weight, FFM, HC, physical activity, and blood pressure levels. However, DOR patients exhibited a higher FM compared to controls (38.47 vs. 36.47; *p* = 0.02). Additionally, women with DOR had significantly greater WC (102.23 vs. 91.7; *p* = 0.002) and WHR (0.9 vs. 0.86; *p* = 0.003) compared to the controls. The DOR group had significantly lower levels of AMH (0.56 vs. 4.11) and AFC values (2.34 vs. 9.59) compared to the control group, showing statistical significance (*p* < 0.001) in both comparisons. Although cases and controls were frequency‐matched according to age and BMI at the study‐design stage, the re‐analysis showed a statistically significant difference in BMI between the groups (*p* < 0.001). This residual difference was accounted for by including BMI as a covariate in the multivariable logistic regression models.

**Table 1:** Baseline characteristics of participants.

Variable		Case (*N *= 120)	Control (*N *= 250)	*p* value^a^
Age (years)		33.37 ± 3.24	32.91 ± 3.15	0.196
BMI (kg/m^2^)		29.85 ± 2.49	27.75 ± 3.45	< 0.001
Weight (kg)		80.96 ± 4.78	79.26 ± 8.41	0.487
FM (kg)		38.47 ± 7.05	36.47 ± 8.91	0.020
FFM (kg)		57.99 ± 11.33	60.12 ± 11.97	0.098
WC (cm)		102.23 ± 35.95	91.70 ± 12.43	0.002
HC (cm)		109.10 ± 31.59	106.10 ± 11.57	0.316
WHR		0.90 ± 0.12	0.86 ± 0.08	0.003
SBP (mmHg)		122.18 ± 12.77	123.58 ± 14.03	0.341
DBP (mmHg)		79.41 ± 11.67	81.85 ± 10.48	0.056
Physical activity (MET/h/day)		19.05 ± 4.12	18.98 ± 4.51	0.896
Socioeconomic status (SES) (%)	Low	10 (8.30)	19 (7.60)	0.252
	Middle	50 (41.70)	127 (50.80)	
	High	60 (50.00)	104 (41.60)	
Education (%)	Illiterate	14 (11.70)	34 (13.60)	< 0.001
	≤ High school/diploma	31(25.80)	121 (48.40)	
	≥ College degree	75 (62.50)	95 (38.00)	
Occupation (%)	Housewife	82 (68.30)	184 (73.60)	< 0.001
	Employed	26 (21.70)	10 (4.00)	
	Student	12 (10.00)	56 (22.40)	
Pervious pregnancy	Yes	99 (82.50)	203 (81.20)	0.441
	No	21 (17.50)	47 (18.80)	
AFC count		2.34 ± 1.19	9.59 ± 2.24	< 0.001
AMH (ng/mL)		0.56 ± 0.71	4.11 ± 1.18	< 0.001

*Note:* Quantitative variables are expressed as mean ± SD and qualitative variables expressed as *n* (%).

Abbreviations: AFC, antral follicle count; BMI, body mass index; DBP, diastolic blood pressure; DOR duration, diminished or decreased ovarian reserve; FFM, fat free mass; FM, fat mass; HC, hip circumference; SBP, systolic blood pressure; WC, waist circumference; WHR, waist‐to‐hip ratio.

^a^ *p* values resulted from independent *t*‐tests for quantitative and *χ* ^2^ for qualitative variables between the two groups.

Table 2 shows the distribution of participants in energy‐adjusted DASH score tertiles, according to main characteristics. Participants in the highest energy‐adjusted DASH tertile had significantly higher AFC count (*p* = 0.015) compared to those in the lowest tertile. DBP level of DOR patients was significantly lower in the third tertile compared to the first tertile (74.84 vs. 80.11; *p* = 0.006). No significant differences were observed in other variables such as age, BMI, WC, WHR, and physical activity among the tertiles of energy‐adjusted DASH score.

**Table 2:** Characteristics of study participants according to tertiles of DASH.

Variable		Case (120)				Control (250)			
		T1	T2	T3	*p* *	T1	T2	T3	*p* *
Age (years)		33.30 ± 2.83	33.84 ± 4.02	32.97 ± 2.75	0.497	33.15 ± 3.23	32.65 ± 3.07	32.93 ± 3.16	0.611
BMI (kg/m^2^)		30.30 ± 3.04	29.54 ± 2.12	29.64 ± 2.13	0.333	27.51 ± 3.66	27.38 ± 3.38	28.30 ± 3.28	0.168
Weight (kg)		82.69 ± 4.42	81.76 ± 3.94	82.52 ± 3.89	0.564	78.57 ± 4.71	77.58 ± 5.90	78.34 ± 3.99	0.405
FM (kg)		36.63 ± 4.86	39.66 ± 8.75	39.33 ± 6.93	0.099	37.34 ± 8.66	37.43 ± 8.70	34.83 ± 9.19	0.095
FFM (kg)		59.25 ± 11.76	58.22 ± 12.07	56.32 ± 10.08	0.508	59.01 ± 11.43	57.83 ± 11.21	63.23 ± 12.59	0.007
WC (cm)		95.76 ± 32.59	105.07 ± 37.34	106.63 ± 37.97	0.334	91.88 ± 11.71	91.79 ± 13.09	91.46 ± 12.57	0.973
HC (cm)		100.32 ± 19.15	113.23 ± 35.41	114.78 ± 36.96	0.072	106.20 ± 10.32	106.40 ± 11.58	105.74 ± 12.69	0.929
WHR		0.89 ± 0.12	0.89 ± 0.10	0.92 ± 0.14	0.461	0.86 ± 0.07	0.86 ± 0.08	0.86 ± 0.08	0.962
SBP (mmHg)		123.81 ± 12.65	124.25 ± 11.49	118.21 ± 13.53	0.06	122.59 ± 13.81	122.56 ± 14.55	125.39 ± 13.71	0.317
DBP (mmHg)		80.11 ± 11.91	83.10 ± 12.67	74.84 ± 8.70	0.006	82.21 ± 9.59	80.59 ± 12.29	82.69 ± 10.17	0.424
AFC count		1.93 ± 1.14	2.48 ± 1.09	2.65 ± 1.25	0.015	9.68 ± 2.42	9.85 ± 2.05	9.26 ± 2.21	0.214
AMH (ng/mL)		0.52 ± 0.23	0.67 ± 0.19	0.49 ± 0.21	0.494	4.14 ± 1.27	4.06 ± 1.23	4.13 ± 1.07	0.906
Physical activity (MET/h/day)		20.09 ± 3.75	18.48 ± 4.26	18.44 ± 4.24	0.117	19.11 ± 4.34	19.28 ± 4.61	18.60 ± 4.59	0.596
Socioeconomic status (SES) (%)	Low	5 (11.60)	3 (7.70)	2 (5.30)	0.039	5 (6.30)	8 (9.80)	6 (6.70)	0.899
	Middle	11 (25.60)	23 (59.00)	16 (42.10)		39 (49.40)	41 (50.00)	47 (52.80)	
	High	27 (62.80)	13 (33.30)	20 (52.60)		35 (44.30)	33 (40.20)	36 (40.40)	
Education (%)	Illiterate	4 (9.30)	5 (12.80)	5 (11.70)	0.469	13 (16.50)	10 (12.20)	11 (12.40)	0.843
	≤ High school/diploma	9 (20.90)	13 (33.30)	9 (27.30)		38 (48.10)	42 (51.20)	41 (46.10)	
	≥ College degree	30 (69.80)	21 (53.80)	24 (63.20)		28 (35.40)	30 (36.60)	37 (41.60)	
Occupation (%)	Housewife	28 (65.10)	27 (69.20)	27 (71.70)	0.219	58 (73.40)	61 (74.40)	65 (73.00)	0.614
	Employed	10 (23.30)	11 (28.20)	5 (13.20)		1 (1.30)	4 (4.90)	5 (5.60)	
	Student	5 (11.60)	1 (2.60)	6 (15.80)		20 (25.30)	17 (20.70)	19 (21.30)	
Previous pregnancy	No	40 (93.00)	29 (74.40)	30 (78.90)	0.067	63 (79.70)	68 (82.90)	72 (80.90)	0.872
	Yes	3 (7.00)	10 (25.60)	8 (21.10)		16 (20.30)	14 (17.10)	17 (19.10)	

*Note:* Quantitative variables are expressed as mean ± SD and qualitative variables expressed as *n* (%). The SES score was evaluated based on the education level of both subjects and the family head, job of both subjects and the family head, family size, home status, and home type by using self‐reported questionnaire.

Abbreviations: AFC, antral follicle count; BMI, body mass index; DBP, diastolic blood pressure; DOR duration, diminished or decreased ovarian reserve; FFM, fat free mass; FM, fat mass; HC, hip circumference; SBP, systolic blood pressure; WC, waist circumference; WHR, waist‐to‐hip ratio.

* *p* values resulted from the ANOVA test for quantitative and *χ* ^2^ for qualitative variables across quartiles.

Table 3 presents the crude and multivariable‐adjusted ORs and 95% CIs for DOR across tertiles of the energy‐adjusted DASH score. No statistically significant association was observed between DASH adherence and the odds of DOR. In the crude analysis, compared with participants in the lowest tertile (T1), the odds of DOR were 13% lower in T2 (OR: 0.87; 95% CI: 0.51–1.49) and 22% lower in T3 (OR: 0.78; 95% CI: 0.46–1.33); however, the linear trend across tertiles was not statistically significant (P‐trend = 0.370). After adjustment for potential confounders, the estimates remained statistically non‐significant in Model 1 (T2: OR: 0.93; 95% CI: 0.53–1.63; T3: OR: 0.76; 95% CI: 0.43–1.33; P‐trend = 0.328) and Model 2 (T2: OR: 0.88; 95% CI: 0.49–1.56; T3: OR: 0.74; 95% CI: 0.42–1.30; P‐trend = 0.288). Besides, when the DASH score was analyzed as a continuous variable, each one‐unit increase in the DASH score was not significantly associated with the odds of DOR in the fully adjusted model (OR = 0.99, 95% CI: 0.93–1.04, *p*= 0.628). The results of the crude, Model 1, and Model 2 analyses are presented in Supporting Information S1: Table S1. Sensitivity analyses that additionally accounted for education and occupation produced findings consistent with the primary analyses. The estimated associations between DASH adherence and the odds of DOR were similar in magnitude and direction to those observed in the primary models, with no statistically significant associations across DASH tertiles (Supporting Information S1: Table S2).

**Table 3:** Odds ratio (95% CI) of DOR according to tertiles of DASH.

	DASH			
	T1	T2	T3	P‐trend
DOR/control	43/79	39/82	38/89	
Crude	Ref (1.00)	0.87 (0.51–1.49)	0.78 (0.46–1.33)	0.370
Model 1	Ref (1.00)	0.93 (0.53–1.63)	0.76 (0.43–1.33)	0.328
Model 2	Ref (1.00)	0.88 (0.49–1.56)	0.74 (0.42–1.30)	0.288

*Note:* Multivariable logistic regression models were used with adjustment of potential confounders. Model 1: Physical activity + age + body mass index (BMI). Model 2: Model 1 + fat mass (kg) and energy intake (kcal/ day).

Abbreviation: DOR, diminished or decreased ovarian reserve.

## 4 Discussion

To our knowledge, this case‐control study is the first to assess dietary quality assessed by energy‐adjusted DASH score in relation to the odds of DOR and its biomarkers (AMH and AFC). In this sample of Iranian women, adherence to the DASH dietary pattern was not significantly associated with the odds of DOR in either crude or multivariable‐adjusted analyses. These findings, therefore, do not provide sufficient evidence to support an association between DASH adherence and the likelihood of DOR in this population. Nevertheless, in an exploratory, unadjusted analysis among women with DOR, AFC differed significantly across energy‐adjusted DASH tertiles; however, this finding should be interpreted cautiously.

Notably, the DASH dietary pattern represents a comprehensive dietary approach characterized by a high intake of fruits, vegetables, whole grains, legumes, nuts, and low‐fat dairy products, together with limited consumption of sodium, processed foods, and saturated fats. Although our findings did not demonstrate a statistically significant association between adherence to the DASH diet and the odds of DOR, the potential relevance of this dietary pattern to reproductive health may be supported by its established beneficial effects on several metabolic and physiological pathways. Previous studies have reported that adherence to the DASH diet is associated with better cardiometabolic [26] and bone health [27] and a lower risk of Type 2 diabetes [28] and cardiovascular diseases [29]. More broadly, reducing the population burden of modifiable cardiometabolic risk factors through dietary and lifestyle strategies has been emphasized as an important public health priority [30]. Interestingly, following a DASH diet for 12 weeks unveiled some favorable influences on body weight, oxidative stress, insulin metabolism, and AMH levels in women with PCOS [18]. These findings provide biological plausibility for a potential relationship between overall dietary quality and reproductive health; however, they cannot be considered direct evidence of an association between DASH adherence and DOR.

Similarly, our previous investigation showed that higher adherence to Diet Quality Index–International (DQI‐I) was associated with lower odds of DOR, with each one‐unit enhancement in DQI‐I score corresponding to a 6% reduction in the odds of DOR [31]. Another investigation among women with DOR reported an inverse association between the antioxidant capacity of the diet and infertility after adjustment for potential confounding factors [32]. Although these findings are consistent with the hypothesis that overall diet quality and antioxidant‐rich dietary patterns may be relevant to reproductive health, differences in dietary assessment methods, study populations, and outcome definitions should be considered when comparing these findings with the present results. Importantly, the absence of a statistically significant association between DASH adherence and DOR in our study indicates that these findings should not be extrapolated to suggest a specific protective effect of the DASH dietary pattern against DOR.

Consistent with the exploratory observation regarding AFC, a cross‐sectional study reported that adherence to a dietary pattern characterized by higher consumption of whole grains, soy, seafood, dairy products, and fruits and vegetables with low pesticide residues was associated with higher AFC among women with obesity [33]. Besides, they reported a similar significant relation for AMH levels, which was not observed in our study [33]. Conversely, a prospective cohort study conducted among women attending a fertility center found no significant association between three healthy dietary patterns, namely the Mediterranean diet, fertility diet, and profertility diet, and AFC [34]. These discrepant findings may partly reflect differences in study populations, dietary assessment methods, definitions, and diagnostic criteria for diminished ovarian reserve, laboratory assays used to assess ovarian reserve biomarkers [35], and study design. In addition, differences in participant characteristics and the presence of residual confounding may contribute to the observed heterogeneity across studies. Therefore, further prospective studies using standardized assessments of dietary intake and ovarian reserve biomarkers are warranted to clarify whether specific dietary patterns are associated with ovarian reserve and whether such associations differ according to metabolic or reproductive characteristics.

One of the secondary findings of the present study was that, among women with DOR, those in the highest tertile of the energy‐adjusted DASH score had significantly lower DBP than those in the lowest tertile. Although this observation is consistent with previous evidence supporting an association between adherence to the DASH dietary pattern and improved blood pressure control [36], this analysis was exploratory and should be interpreted with caution. A recent comprehensive review has demonstrated that the DASH diet is an effective dietary strategy for blood pressure management, which has been attributed to its emphasis on fruits, vegetables, whole grains, low‐fat dairy products, and other nutrient‐rich foods that provide potassium, magnesium, calcium, fiber, and protein, while limiting sodium, saturated fat, and added sugars [36]. Nevertheless, because blood pressure was not a primary outcome of the present study, further investigations specifically designed to evaluate the association between adherence to the DASH diet and blood pressure among women with DOR are warranted.

Although the precise biological mechanisms linking adherence to the DASH dietary pattern with ovarian function remain unclear, several components of this dietary pattern may plausibly influence metabolic and inflammatory pathways relevant to reproductive health. Previous evidence suggests that adherence to the DASH diet may be associated with improved insulin sensitivity and favorable alterations in inflammatory and oxidative stress pathways [26], which have been implicated in impaired reproductive function [37]. Moreover, the DASH dietary pattern has been recognized as a potentially beneficial dietary approach for weight management and prevention of obesity [38], which may be relevant to reproductive health given the established relationship between excess adiposity and fertility‐related outcomes [39].

In addition, the relatively high consumption of low‐glycemic‐index foods within a DASH‐oriented dietary pattern may contribute to improved insulin sensitivity and glucose homeostasis [40]. These metabolic effects are of potential relevance to reproductive function, particularly because insulin resistance and disturbances in glucose metabolism have been implicated in anovulatory infertility. Nevertheless, these proposed pathways represent biologically plausible mechanisms and should not be interpreted as evidence of a direct effect of DASH adherence on ovarian reserve. Given that we did not observe a statistically significant association between DASH adherence and the odds of DOR in the present study, further prospective and mechanistic investigations are warranted to determine whether, and through which pathways, dietary patterns may influence ovarian reserve and reproductive function.

Among the strengths of the present study are providing novel insight into the relationship of energy‐adjusted DASH score and odds of DOR along with ovarian markers; a large sample size, which produces a more accurate estimation of relations; the consideration of age and BMI in the study design; and controlling for potential confounders. Nevertheless, this investigation had several limitations that should be noted. First, owing to the retrospective case‐control design, the temporal relationship between adherence to the DASH diet and DOR cannot be established; therefore, causal inferences cannot be drawn. Second, dietary intake was assessed using a validated semi‐quantitative FFQ that evaluated habitual dietary intake over the preceding year. Nevertheless, because dietary information was self‐reported, recall bias cannot be completely excluded. Moreover, reverse causation is possible, as women experiencing fertility concerns may have modified their dietary habits before study enrollment, potentially influencing the observed associations. Third, the influence of unmeasured or residual confounding factors, such as genetic background and psychological factors, cannot be completely excluded. Besides, although cases and controls were frequency‐matched for age and BMI, a significant residual difference in BMI remained between the groups. BMI was therefore included in the multivariable models to minimize potential residual confounding; however, the possibility of residual confounding cannot be completely excluded. Finally, because this was a hospital‐based study conducted among women attending an infertility clinic, the generalizability of our findings to the broader population may be limited, and some degree of selection bias cannot be completely excluded.

In conclusion, higher adherence to the DASH dietary pattern was not significantly associated with the odds of DOR among Iranian women. Nevertheless, the exploratory association observed between DASH adherence and AFC among women with DOR may warrant further investigation into the potential relationship between overall dietary quality and ovarian reserve. Given the retrospective case‐control design of the present study, these findings should be interpreted cautiously and cannot establish temporal or causal relationships. Further prospective cohort studies and well‐designed intervention trials are warranted to clarify whether adherence to the DASH dietary pattern is associated with ovarian reserve and to elucidate the underlying mechanisms.

## Author Contributions

Abed Ghavami and Gholamreza Askari were involved in the conception and design of the work. Hatav Ghasemi‐Tehrani was the study gynecologist and designed the project. Abed Ghavami, Mahdi Vajdi, and Amin Mokari‐Yamchi performed statistical analyses and interpretation of data. Mahdieh Khodarahmi, Sanaz Mehrabani, and Abed Ghavami drafted the work and revised it.

## Ethics Statement

All procedures were approved by the Ethics Committee of Isfahan University of Medical Sciences (IR.ARI.MUI.REC.1401.297) and were conducted in accordance with the Declaration of Helsinki.

## Consent

Written informed consent was obtained from all participants prior to enrollment.

## Conflicts of Interest

The authors declare no conflicts of interest.

## Transparency Statement

The corresponding author, Abed Ghavami, affirms that this manuscript is an honest, accurate, and transparent account of the study being reported; that no important aspects of the study have been omitted; and that any discrepancies from the study as planned (and, if relevant, registered) have been explained.

## Supporting information


Supporting File

## Data Availability

The data supporting the findings of this study are available from the corresponding author upon reasonable request.
