# Supplementary material for: Association Between Adherence to the Dietary Approaches to Stop Hypertension (DASH) Diet and Diminished Ovarian Reserve: A Case–Control Study
Source: Health Sci Rep. 2026 Nov 3;9(11):e73457. doi: 10.1002/hsr2.73457 (PMC13729006; doi:10.1002/hsr2.73457)
Supplement: Supplementary file 1 [file HSR2-9-e73457-s001.docx]

**Table S1. Association between DASH score as a continuous variable and odds of DOR**

| **DASH score (per 1-unit increase)** | **Crude OR (95% CI)** | **Model 1 OR (95% CI)** | **Model 2 OR (95% CI)** |
| --- | --- | --- | --- |
| **Continuous DASH score** | 0.99 (0.95-1.05) | 0.99 (0.94-1.04) | 0.99 (0.93-1.04) |
| **P-value** | 0.818 | 0.706 | 0.628 |

|  | DASH | | | |
| --- | --- | --- | --- | --- |
|  | T1 | T2 | T3 | P-trend |
| DOR/Control | 43/79 | 39/82 | 38/89 |  |
| **Crude** | Ref (1.00) | 0.87 (0.51-1.49) | 0.78 (0.46-1.33) | 0.370 |
| **Model 1** | Ref (1.00) | 0.86 (0.47–1.59) | 0.76 (0.42–1.38) | 0.361 |
| **Model 2** | Ref (1.00) | 0.82 (0.44–1.52) | 0.73 (0.40-1.34) | 0.311 |

**Table S2. Odds Ratio (95% CI) of DOR According to Tertiles of DASH**

Multivariable logistic regression models were used with adjustment of potential confounders. Model 1: Physical Activity + Age + Body mass index (BMI) + Education + Occupation. Model 2: Model 1 + Fat Mass (kg) and Energy intake (kcal/ day). Abbreviation: DOR: diminished or decreased ovarian reserve.
